# Familiarity and concentration- based intensity shape spatial patterns of odor-evoked brain activation

**DOI:** 10.1186/s12993-026-00375-5

**Published:** 2026-11-05

**Authors:** Akshita Joshi, Sophia Frieling, Divesh Thaploo, Antje Haehner, Anne Abriat, Thomas Hummel

**Affiliations:** 1 Faculty of Medicine Carl Gustav Carus, Department of Otorhinolaryngology, Smell and Taste Clinic, Technische Universität Dresden, Fetscherstraße 74, 01307 Dresden, Germany; 2 National Smell and Taste center, Division of Intramural Research, National Institute on Deafness and Other Communication Disorders (NIDCD), National Institutes of Health, Bethesda, MD USA; 3 The Smell and Taste Lab, Rue Cramer 6, Genève, 1202 Switzerland

**Keywords:** Olfactory perception, Odor familiarity, Perceptual intensity, Odor-evoked memory, fMRI

## Abstract

Olfactory perception is shaped by both the physical properties of odorants and prior odor experience. Odor familiarity and concentration-based perceptual intensity are known to influence neural responses; however, less is known about how odor-evoked brain activation varies across conditions that differ in familiarity and perceived strength. Using functional magnetic resonance imaging, fMRI, we examined condition-specific activation patterns and brain-behavior relationships during passive odor perception. Sixty-three healthy young women participated in a 2 × 2 factorial fMRI experiment including familiar and unfamiliar odors presented at low- and high-intensity concentrations. Odors were delivered birhinally using a computer-controlled olfactometer, and participants provided in-scanner ratings of perceived odor intensity and odor-evoked memory intensity. Whole-brain analyses were used to describe condition-wise activation maps and direct between-condition contrasts, while region-of-interest analyses focused on canonical olfactory, limbic, and memory-related regions. All odor conditions elicited activation across olfactory, limbic, frontal, parietal, and cerebellar regions. Direct contrasts showed that low-intensity unfamiliar odors elicited greater activation than low-intensity familiar odors in frontal and parietal regions, suggesting increased evaluative processing when weak odor input could not be readily matched to established odor representations. Low-intensity unfamiliar odors also elicited greater activation than high-intensity unfamiliar odors in precuneus and supramarginal/angular regions. Brain-behavior analyses showed significant associations only for familiar odors presented at low intensity, where subjective memory intensity was negatively correlated with bilateral parahippocampal activation. These findings suggest that odor familiarity and concentration-based intensity contribute to condition-specific patterns of odor-evoked brain activation, with low-intensity familiar odors revealing a selective relationship between odor-evoked memory experience and medial temporal responses.

**Supplementary Information:**

The online version contains supplementary material available at https://doi.org/10.1186/s12993-026-00375-5.

## Introduction

Olfactory perception is inherently shaped by both the physical properties of odorants and the associative meaning they carry [39, 11]. Among the most influential factors is odor familiarity, the extent to which an odor has been encountered previously and incorporated into memory. Decades of behavioral and neuroimaging research have shown that familiar odors are detected and identified more rapidly than unfamiliar ones and often evoke strong affective and autobiographical responses [33]. These responses typically engage not only primary olfactory regions such as the piriform cortex, but also limbic and paralimbic structures, including the amygdala, hippocampus, and orbitofrontal cortex, which support emotional appraisal and episodic memory [4, 29]. By contrast, unfamiliar stimuli have been reported to elicit greater attentional and evaluative processing, reflected in the activation of fronto-parietal regions that mediate novelty detection and decision-making [22]. This distinction underscores that olfactory perception is a dynamic interplay between sensory input and stored representations of prior experience [34].

A second fundamental dimension of olfactory perception is odor intensity, which refers to the perceived strength of an odor and is commonly influenced by odorant concentration. Perceived intensity is central to olfactory experience because it determines how strongly an odor is represented and how readily it engages downstream sensory, cognitive, and affective processes [13, 16, 28]. Many psychophysical and neuroimaging studies have contrasted undetectable or weakly detectable odors with clearly perceived odors to understand how olfactory signals are represented in the brain [10, 35]. However, in daily life, odors are typically consciously perceived and vary in the strength and vividness of their perceptual impact [39, 43]. The present study therefore compared low-intensity odors, which were weak but detectable, with high-intensity odors, which were perceived as stronger.

Literature suggests that both odor familiarity and intensity influence the engagement of neural systems beyond primary olfactory regions [13, 14]. For example, high-intensity odors have been shown to enhance activation not only in the piriform cortex but also in insula and anterior cingulate cortex, regions implicated in interoceptive awareness and intensity detection [15, 20]. Familiar odors, even at moderate intensity, often engage amygdala, hippocampus, and medial orbitofrontal cortex, consistent with their affective and autobiographical significance [19, 26]. However, most prior studies have considered familiarity and intensity separately, and less is known about how odor-evoked brain activation varies across conditions that differ in prior odor experience and perceived odor strength. This gap is important because odor perception in daily life is shaped by both the physical intensity of an odor and the personal or cultural significance it carries [17]. Examining familiarity and intensity within the same experimental framework can help clarify their respective contributions to spatial patterns of odor-evoked brain activation.

It remains unclear whether familiar and unfamiliar odors recruit different neural systems when perceived at lower or higher intensity levels. For example, unfamiliar odors may require greater evaluative processing because they cannot be easily matched to stored odor representations, whereas familiar odors may engage memory-related regions more strongly, particularly when the sensory input is weak. Similarly, higher-intensity odors may evoke stronger sensory-driven activation regardless of familiarity. Examining these condition-specific patterns can help clarify how stimulus-driven and experience-dependent factors contribute to olfactory brain activation.

Functional magnetic resonance imaging (fMRI) provides a powerful tool for disentangling the contributions of familiarity and intensity to olfactory perception by enabling the whole-brain mapping of neural responses to controlled odor stimuli. Prior neuroimaging studies have demonstrated that olfactory cortical responses scale with intensity and that emotional and autobiographical aspects of familiar odors recruit hippocampal-limbic circuits [27, 45].

The present study aimed to characterize how odor familiarity and concentration-based perceptual intensity contribute to condition-specific spatial patterns of odor-evoked brain activation during olfactory perception. We examined activation patterns across familiar and unfamiliar odors presented at low and high intensity levels. We hypothesized that familiar odors would be associated with greater engagement of olfactory-limbic and memory-related regions, reflecting access to established odor representations. In contrast, unfamiliar odors were expected to recruit evaluative and attentional regions, particularly under low-intensity conditions, where weaker sensory input may require additional interpretive processing. We further expected higher-intensity odors to elicit stronger sensory-driven activation than lower-intensity odors. Finally, we explored whether subjective odor-evoked memory intensity would be associated with activation in memory-related regions, particularly for familiar odors presented at low intensity.

Testing these predictions may help clarify how prior odor experience and concentration-based perceptual intensity contribute to condition-specific patterns of olfactory brain activation.

## Methods

### Participants

A total of 63 healthy young women (mean age = 24.21 ± 3.56 years) participated in the study after providing written informed consent. The study protocol was approved by the Ethics Committee of the Medical Faculty, Technical University of Dresden (approval no. BO-EK-170042022) and conducted in accordance with the Declaration of Helsinki.

All participants were screened to verify eligibility, confirming normal olfactory and gustatory function based on self-report (Smell ability normal? and Taste ability normal?; 1 = yes, 2 = no). Baseline demographic information, including age, weight, and height, was recorded. Each participant then completed a comprehensive battery of validated psychophysical assessments. Healthy and normal olfactory performance was evaluated using the Sniffin’ Sticks subtests for odor threshold and odor identification [30], together with the Sniffin’ TOM test [7], which provided measures of Hits, Correct Rejections, False Alarms, and Unrecognized odors, reflecting odor memory accuracy and recognition performance. Participants also completed the ADS-L questionnaire to evaluate mood and affective state, acknowledging the potential influence of emotional status on olfactory perception and task engagement. Exclusion criteria comprised a history of olfactory dysfunction, psychiatric illness, chronic medical conditions, regular alcohol use, ongoing medication other than hormonal contraception/thyroid hormones, and any history of major neurological disorders. All participants were right-handed and had no contraindications for MRI.

### Odor selection, pilot testing, and finalization

Prior to fMRI scanning, participants completed a laboratory-based odor rating session to determine individualized familiar and unfamiliar odor stimuli. Sixteen commercial perfume-based odorants were presented on fragrance blotter strips, similar to those used in perfumery testing. The odorants consisted of complex fragrance mixtures, with base odor families including floral, woody, and citrus. These stimuli were selected to provide clearly perceivable, naturalistic, and everyday odor percepts rather than single-molecule odorants.

Each participant rated all 16 odorants for familiarity on a numerical scale ranging from 0, not familiar at all, to 10, extremely familiar. Odors with familiarity ratings ≤ 3 were categorized as unfamiliar, whereas odors with familiarity ratings ≥ 7 were categorized as familiar. In the pilot sample, the selected unfamiliar odors had a mean familiarity rating of 1.50 ± 1.00, whereas the selected familiar odors had a mean familiarity rating of 8.25 ± 1.14. Based on these ratings, one familiar odor and one unfamiliar odor were selected individually for each participant and used during fMRI scanning. Thus, familiarity was defined at the participant level rather than assigned uniformly across the full sample.

The selected odorants were subsequently evaluated in an independent pilot study of 12 participants who were not included in the MRI cohort. The purpose of the pilot study was to establish two concentration-based perceptual intensity levels suitable for repeated presentation during scanning. Odors were diluted in propylene glycol to create low-intensity and high-intensity conditions. The low-intensity condition consisted of 10% odorant and 90% propylene glycol, whereas the high-intensity condition consisted of 40% odorant and 60% propylene glycol. These dilution ratios were selected to produce clearly perceivable but distinguishable intensity levels, while minimizing lower-bound effects at low intensity and avoiding sensory saturation or irritation at high intensity.

The pilot study confirmed that the two concentration levels differed significantly in perceived intensity, *p* < 0.001, while preserving the intended familiar or unfamiliar character of the selected odorants. On the day of scanning, participants completed a brief yes/no familiarity check and were reintroduced to their selected odorants to confirm that the assigned familiar and unfamiliar stimuli remained consistent with their subjective familiarity ratings before fMRI acquisition.

### Experimental paradigm

During fMRI scanning, participants completed a passive odor perception task. They were instructed to remain still, keep their eyes open, and fixate on a centrally presented cross while odor stimuli were delivered birhinally. No additional visual cues indicating odor onset were presented, in order to minimize visual-attentional confounds during olfactory stimulation.

The experiment followed a 2 × 2 factorial design with two levels of familiarity, familiar versus unfamiliar, and two levels of concentration-based perceptual intensity, low versus high. This resulted in four functional runs: low-intensity familiar, low-intensity unfamiliar, high-intensity familiar, and high-intensity unfamiliar (low- and high-intensity labels refer to concentration-based intensity conditions). Odors were presented in blocks pattern corresponding to these four experimental conditions. The order of the four runs was pseudo-randomized across participants to minimize order and habituation effects.

Within each run, odorized air was delivered for 2 s, followed by 2 s of odorless air. This 2 s odor/2 s clean-air sequence was repeated twice, resulting in an 8-s of odor-stimulation. Each 8-s odor stimulation was followed by a 12-s odorless-air interval to reduce olfactory adaptation and allow partial recovery before the next stimulation period. Thus, each stimulation cycle lasted 20 s, and this cycle was repeated 10 times per odor condition.

Following each block of odor presentations, participants provided in-scanner ratings of perceived odor intensity and the intensity of any memory evoked by the odor, hereafter referred to as memory intensity. Ratings were provided using numerical scales ranging from 0, not at all, to 10, extremely.

Odors were delivered using a computer-controlled olfactometer [38] positioned outside the scanner room and connected to the participant via Teflon™ tubing. To minimize contamination and ensure stable stimulus delivery, the olfactometer alternated odorized and clean air streams and employed a continuous-flow system. No respiration belt was worn during scanning; therefore, odor delivery was not respiration-triggered. Instead, repeated short odor pulses, clean-air intervals, 12-s odorless-air periods, and continuous airflow were used to provide stable odor perception while reducing adaptation and contamination across repeated presentations.

### Imaging acquisition

All MRI data were acquired on a 3 T MRI scanner (model “Prisma,” Siemens Medical Systems, Erlangen, Germany) using a 32-channel head coil. Functional images were obtained with a T2-weighted single-shot echo-planar imaging (EPI) sequence*: TR = 1000 ms, TE = 40 ms, flip angle = 60°, slice thickness = 3 mm, no inter-slice gap, in-plane resolution 3 × 3 mm with a 64 × 64 matrix (field of view ≈ 192 × 192 mm). Parallel imaging (GRAPPA factor = 2) and multiband acceleration (factor = 3) were used to increase temporal resolution. The pixel bandwidth was 2520 Hz/px, and the phase-encoding direction was along the column axis.

High-resolution anatomical images were acquired using a 3D magnetization-prepared rapid gradient echo (MPRAGE) sequence (TR = 2000 ms, TE = 1.97 ms, voxel size = 1 × 1 × 1 mm³, field of view = 256 × 256 mm²).

### Image preprocessing and statistical analysis

Functional MRI data were preprocessed and analyzed using FSL v6.0.2 [18], employing the FEAT (FMRI Expert Analysis Tool) for subject-level analyses and the gFEAT framework for group-level analyses. Preprocessing steps included motion correction using MCFLIRT to adjust for head movement, slice-timing correction for interleaved acquisition, spatial smoothing with a Gaussian kernel of 6 mm FWHM, and high-pass temporal filtering with a cut-off of 100 s to remove low-frequency drifts. Brain extraction of both structural and functional images was performed using the BET (Brain Extraction Toolbox) [37], and all extracted images were visually inspected with thresholds adjusted as needed to minimize inclusion of non-brain tissue while preserving cortical structures. Scans with severe motion artifacts or unresolvable signal dropout were excluded from further analysis.

At the first-level analysis, data from each participant and each of the four experimental runs (low-intensity familiar, low-intensity unfamiliar, high-intensity familiar, high-intensity unfamiliar) were modeled using a general linear model (GLM) with task-related regressors convolved with a canonical double-gamma hemodynamic response function, while six motion parameters were included as nuisance regressors. At the second-level (within-subject) analysis, parameter estimates from the four runs were combined using a fixed-effects model to obtain subject-specific contrasts. At the group level analysis, we first generated a group-mean activation map to identify overall task-related activations across all participants. We then applied GLM-based contrasts to test for the main effects of familiarity (e.g., low-intensity familiar vs. low-intensity unfamiliar: [1–1] and [-1 1]) and concentration- based intensity (e.g., low vs. high intensity within familiar or unfamiliar conditions). To account for individual differences in olfactory-related symptoms, ADSL scores were included as a covariate of no interest in the group-level GLM to control for their potential influence on neural activation patterns. Group-level statistical modeling was performed using FLAME1 (FMRIB’s Local Analysis of Mixed Effects; [44], which accounts for both within-subject and between-subject variance to yield robust population-level estimates. Whole-brain statistical inference was performed using FSL’s Gaussian random field theory-based cluster correction. Z-statistic images were thresholded using a cluster-forming threshold of Z > 3.1, corresponding to *p* < 0.001 uncorrected at the voxel level, followed by cluster-level correction at *p* < 0.05. Resulting clusters were reported for condition-wise activation maps and direct between-condition contrasts.

### Statistical analysis

Behavioral data were analyzed using GraphPad Prism, version 10. Perceived odor intensity and odor-evoked memory intensity ratings were compared using two-tailed within-subject paired t-tests, because the same participants completed all experimental conditions. Pairwise comparisons were performed to assess intensity-related differences within each familiarity category, high- versus low-intensity familiar odors and high- versus low-intensity unfamiliar odors, and familiarity-related differences within each intensity level, familiar versus unfamiliar odors under high- and low-intensity conditions. One participant was excluded from the behavioral analyses because of incomplete in-scanner rating data; therefore, analysis was conducted using complete data from 62 participants. Behavioral results are reported with the test statistics, degrees of freedom, exact p-value, and Cohen’s dz as the effect size for paired comparisons.

Brain-behavior relationships were assessed using ROI-based correlation analyses. Behavioral measures included in-scanner perceived odor intensity ratings, in-scanner odor-evoked memory intensity ratings, and the trait-level Total Test of Odor Memory, TOM, score, calculated as hits plus correct rejections and obtained once per participant. Neural responses were quantified as region-of-interest, ROI, based percentage signal change, %SC, extracted for each participant from the four experimental conditions: low-intensity familiar, low-intensity unfamiliar, high-intensity familiar, and high-intensity unfamiliar odors.

ROIs were defined a priori based on canonical olfactory and memory-related regions consistently implicated in odor perception and odor-evoked memory processing. The final ROI set included the bilateral hippocampus, parahippocampal gyrus, amygdala, anterior cingulate cortex, piriform cortex, and orbitofrontal cortex. Anatomical ROIs for the hippocampus, parahippocampal gyrus, amygdala, and anterior cingulate cortex were generated using WFU PickAtlas in MNI space, while piriform and orbitofrontal cortex ROIs were obtained from validated, previously published masks [41, 42]. ROIs were warped into each participant’s functional space using FSL, applywarp. Contrast of parameter estimate, COPE, values for the odor on-off contrast were extracted and converted to %SC using the mean functional signal.

For each ROI and experimental condition, Pearson correlations were computed across participants between behavioral measures and ROI %SC values. To assess the robustness of ROI brain-behavior associations and reduce reliance on parametric assumptions, statistical significance was estimated using a two-tailed permutation-based procedure. For each ROI-condition pair, behavioral ratings were randomly permuted across participants 5,000 times, and the correlation coefficient was recalculated for each permutation to generate a null distribution. Empirical two-tailed permutation p-values were calculated as the proportion of permuted absolute correlation coefficients greater than or equal to the observed absolute correlation coefficient. For the memory-intensity analysis, permutation-based p-values were corrected for multiple comparisons across all ROI × condition tests using the Benjamini-Hochberg false discovery rate, FDR, procedure (Table 1).

## Results

**Table 1:** summarizes all demographic and behavioral measures obtained in this study, expressed as mean ± standard deviation (SD)

Variable	Mean ± SD
Age (years)	24.21 ± 3.56
Odor threshold score	6.95 ± 1.29
Odor identification score	13.52 ± 1.13
TOM - Hits	7.25 ± 0.76
TOM - Correct rejections	6.71 ± 1.14
TOM - False alarms	1.32 ± 1.15
TOM - Unrecognized	0.68 ± 0.74
ADSL (Affective/Depression Scale)	15.13 ± 4.28

### Behavioral results

#### Familiar odors

**Fig. 1:**
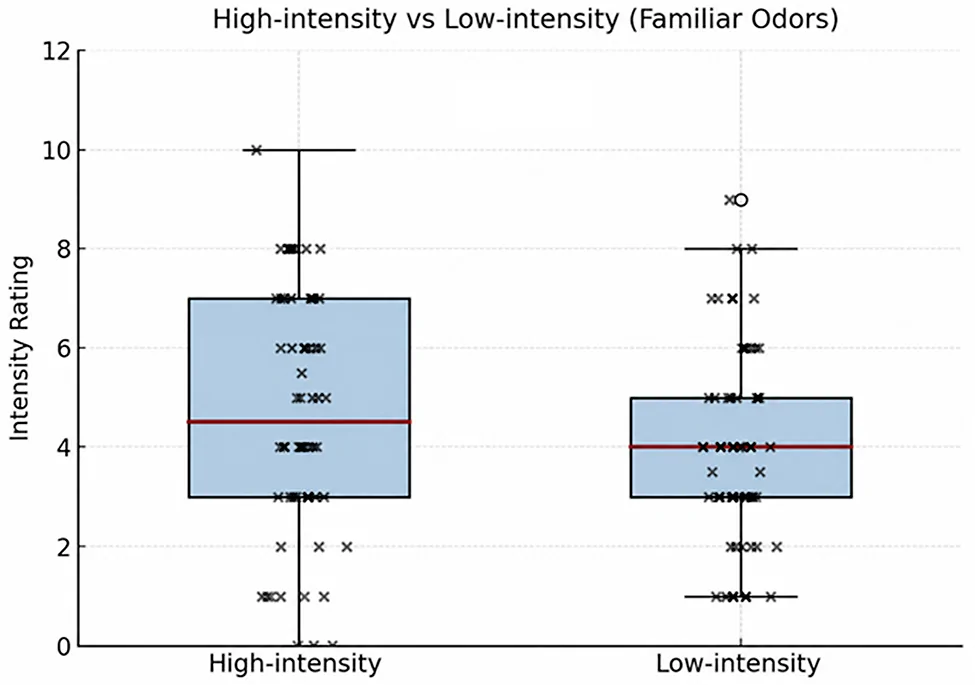
Box plot illustrates perceived odor intensity ratings for familiar odors under high- and low-intensity conditions. Each dot represents an individual participant’s rating. The median is indicated by the red line, and boxes represent the interquartile range (IQR). High-intensity familiar odors were rated as significantly more intense than low-intensity familiar odors, t(61) = 2.10, *p* = 0.040, Cohen’s dz = 0.27

Although 63 participants completed the study, one participant was excluded from this comparison because of incomplete in-scanner rating data; therefore, this analysis included 62 participants with complete paired ratings. For familiar odors, there was a significant difference in perceived intensity between high-intensity and low-intensity conditions, t(61) = 2.10, *p* = 0.040, (Fig. 1) with participants rating high-intensity odors as more intense (M = 4.67) compared to low-intensity odors (M = 4.07). In contrast, no significant difference was observed in memory intensity ratings between high- and low-intensity conditions, t(61) = 1.30, *p* = 0.204, with mean scores of 3.94 and 3.06, respectively.

#### Unfamiliar odors

For unfamiliar odors, there were no significant differences in either perceived intensity or memory intensity between high- and low-intensity conditions. Mean intensity ratings were 4.74 (HI) and 4.34 (LI), t(61) = 1.23, *p* = 0.22 while mean memory intensity ratings were 3.52 (HI) and 2.65 (LI), t(61) = 1.12, *p* = 0.273.

#### Familiar vs. unfamiliar comparisons

When comparing familiar and unfamiliar odors directly, no significant differences existed in either perceived intensity or memory intensity under high- or low-intensity conditions. For the high-intensity condition, intensity ratings did not differ significantly between familiar (M = 4.67) and unfamiliar (M = 4.74) odors, t(61) = − 0.21, *p* = 0.834. Similarly, for the low-intensity condition, intensity ratings were comparable across familiar (M = 4.12) and unfamiliar (M = 4.34) odors, t(61) = − 0.62, *p* = 0.540. For memory intensity, ratings were also similar between familiar and unfamiliar odors under both high intensity - (t(61) = 0.70, *p* = 0.485; M = 4.45 vs. 4.03) and low intensity conditions (t(61) = 0.11, *p* = 0.912; M = 3.08 vs. 3.03).

### Condition-wise odor-evoked activation maps

Condition-wise one-sample activation maps were used to describe odor-evoked activation within each experimental condition. These maps are reported descriptively and should not be interpreted as direct statistical evidence for differences between conditions. Z statistic images were thresholded at *p* < 0.001, uncorrected, with a minimum cluster extent of 20 voxels. Cluster-level details, including voxel extent, Z-maxima, and MNI coordinates, are provided in Supplementary File 1, Table S1-S4.

#### Low-Intensity Familiar Odors

For the low-intensity familiar odor condition, activation was observed bilaterally in the amygdala, parahippocampal gyrus, and orbitofrontal cortex, extending into the insula and piriform regions. Additional activations were found in the middle and inferior frontal gyri, posterior cingulate cortex, and precuneus. Posterior clusters included occipital and cerebellar regions, particularly within the lingual gyrus, cerebellar vermis, and Crus I-II lobules. (Supplementary file 1 Table S1)

#### Low-Intensity unfamiliar odors

For the low-intensity unfamiliar odor condition, significant clusters were identified within frontal, temporal, parietal, and cerebellar regions. Prominent activations were located in the superior, middle, and inferior frontal gyri, encompassing dorsolateral and orbitofrontal areas. Additional clusters were observed in the insula, cingulate cortex, and caudate nucleus, as well as in posterior parietal, precuneus, and occipital cortices. Bilateral cerebellar activations were evident in Lobule VI and Crus I-II, together with responses in the superior temporal gyrus and parahippocampal regions. (Supplementary file 1 Table S2)

#### High-intensity familiar odors

The high-intensity familiar odor condition revealed activations in frontal, temporal, and cerebellar regions. Significant clusters were located in the inferior frontal gyrus and anterior cingulate, extending into prefrontal and orbitofrontal cortices bilaterally. Posterior activations were identified in cerebellar Lobule VI and Crus II, occipital fusiform gyrus, and lingual cortex, with additional foci in superior parietal and precuneus regions. (Supplementary file 1 Table S3)

#### High-intensity unfamiliar odors

For the high-intensity unfamiliar odor condition, widespread bilateral activations were observed across frontal, temporal, parietal, occipital, and cerebellar areas. Significant clusters encompassed the inferior, middle, and superior frontal gyri, including dorsolateral prefrontal, orbitofrontal, and supplementary motor regions. Further activations were noted in the anterior cingulate cortex, insula, frontal operculum, and caudate nuclei. Posteriorly, activation extended into the superior and inferior parietal lobules, precuneus, posterior cingulate, lingual gyrus, and occipital cortex, with strong bilateral clusters in cerebellar Lobule VI, Crus I, and Crus II. (Supplementary file 1 Table S4)

### Direct between-condition contrasts

Direct between-condition contrasts were used to test familiarity- and intensity-related differences in odor-evoked activation. At the group level, Z (Gaussianised T/F) statistic images were thresholded at *p* = 0.001 (uncorrected) with a minimum cluster extent of 20 voxels.

**Fig. 2:**
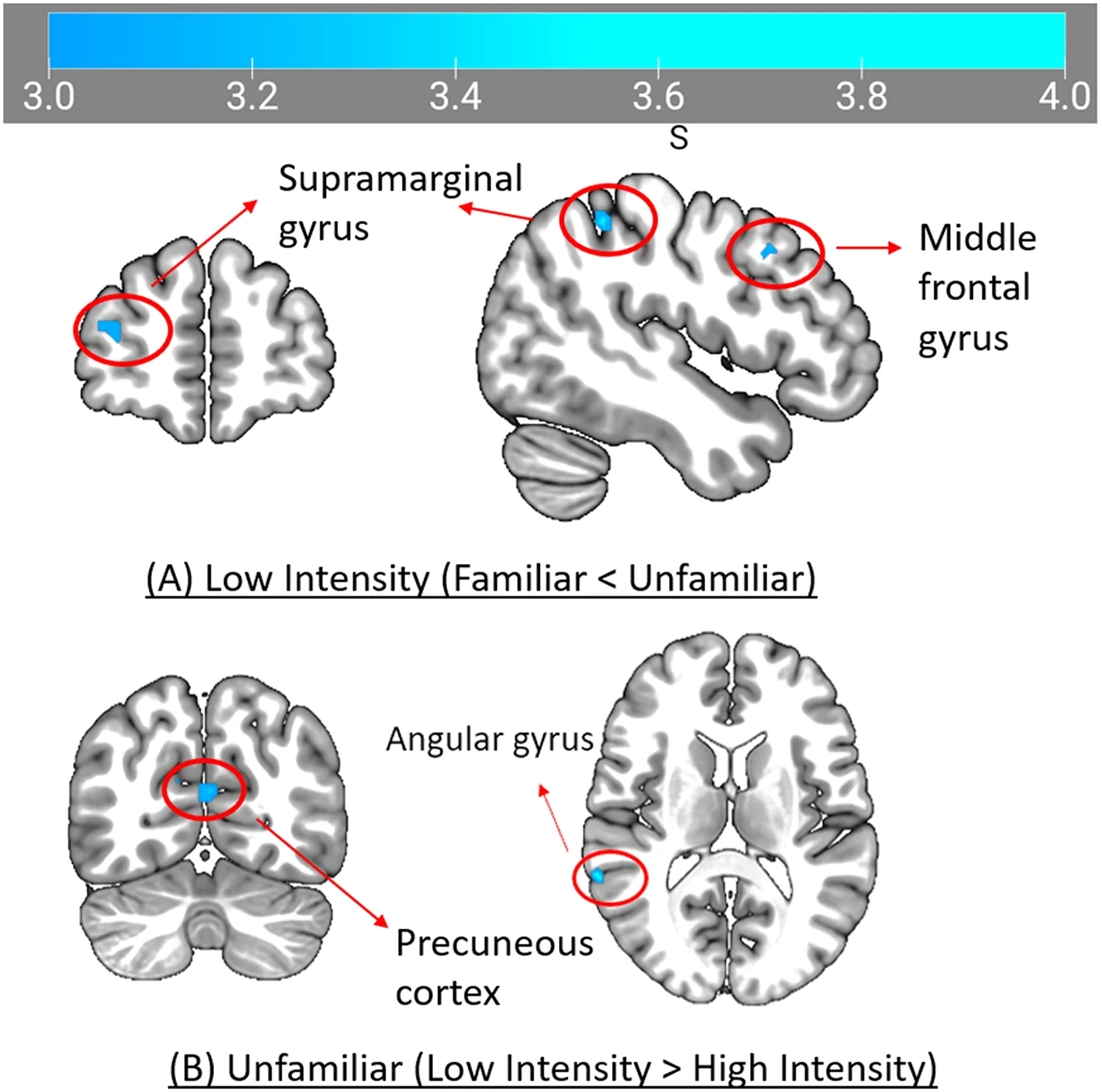
Neural correlates of odor familiarity and concentration-based intensity. Statistical parametric maps illustrate brain regions showing differential activation as a function of odor familiarity and perceptual intensity. (**A**) Low-intensity condition (Familiar < Unfamiliar): Greater responses to unfamiliar relative to familiar odors were observed in the supramarginal gyrus and middle frontal gyrus (highlighted by red circles). (**B**) Unfamiliar odors (Low intensity > High intensity): Enhanced activation for low- compared with high-intensity unfamiliar odors was detected in the precuneus cortex and angular gyrus. Maps are displayed on canonical cortical surfaces and slice views for visualization; the color bar denotes Z-values (3.0–4.0). All clusters are shown at the statistical threshold used in the main analysis

For the low-intensity conditions, the contrast low-intensity familiar < low-intensity unfamiliar revealed significant activations in the right superior frontal gyrus and left superior parietal lobule/postcentral gyrus. The contrast low-intensity unfamiliar > high-intensity unfamiliar showed activation in the precuneus and right middle temporal gyrus. No other contrasts showed activation above threshold (Table 2; Fig. 2).

**Table 2:** Whole-brain activation results for familiarity and intensity effects during odor perception

Contrast	Voxels	Z-MAX	X (mm)	Y (mm)	Z (mm)	Activated Region
Familiar vs. Unfamiliar						
Low-intensity familiar < Low-intensity unfamiliar	40	3.63	36	60	12	Middle frontal gyrus
Low-intensity familiar < Low-intensity unfamiliar	26	3.65	−44	−44	50	Posterior supramarginal gyrus
**Low vs. High Intensity**						
Low-intensity unfamiliar > High-intensity unfamiliar	24	3.43	−2	−68	20	Precuneus
Low-intensity unfamiliar > High-intensity unfamiliar	22	3.75	64	−44	14	Supramarginal gyrus, including angular gyrus

The table summarizes significant clusters from voxel-wise whole-brain analyses, reporting cluster size (voxels), peak Z-statistic (Z-MAX), Montreal Neurological Institute (MNI) peak coordinates (X, Y, Z; mm), and corresponding anatomical regions. Only clusters surviving a voxel-wise threshold of Z > 3.1 with a cluster-forming threshold of *p* < 0.001 (uncorrected) are listed; contrasts with no suprathreshold clusters are not listed in the table

### Brain-behavior correlation analysis

**Fig. 3:**
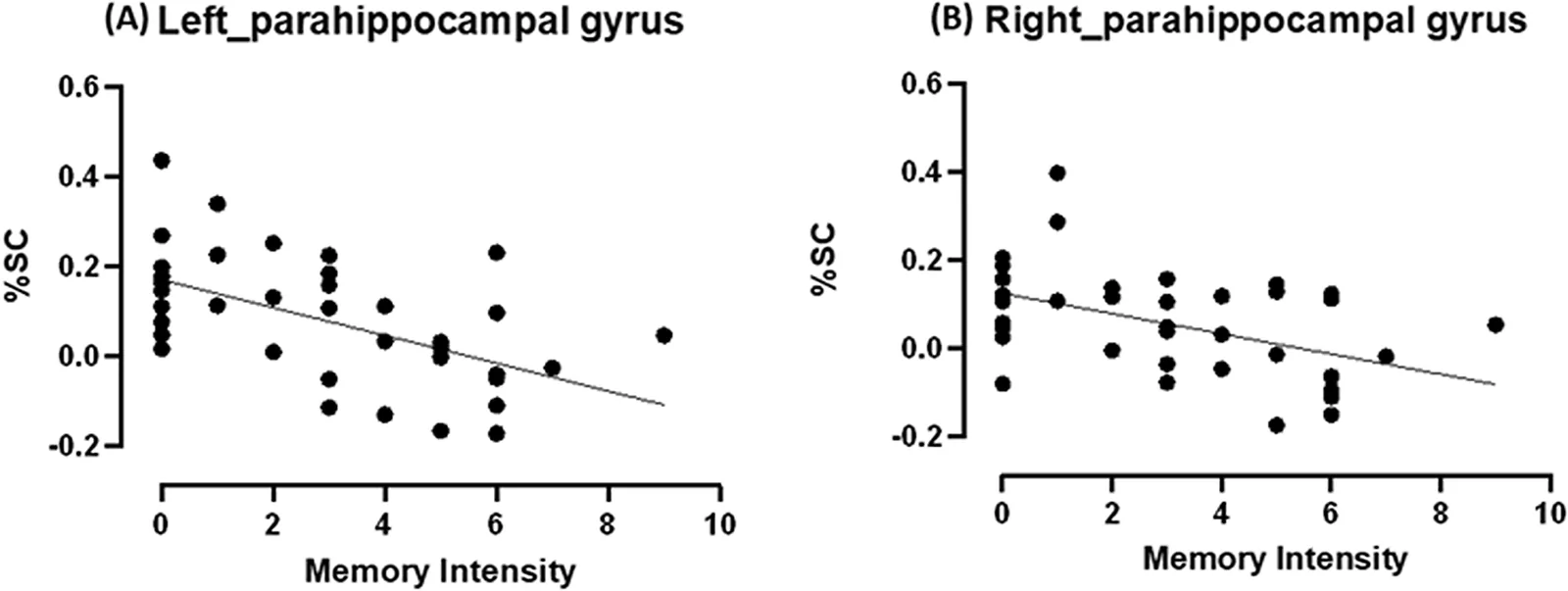
Scatter plots show the relationship between memory intensity ratings (0–10 scale) and percent signal change (%SC) in the (**A**) left and (**B**) right parahippocampal gyrus during the low-intensity familiar odor condition. After false discovery rate (FDR) correction for multiple comparisons, significant negative correlations were observed for both regions (left: *r* = − 0.57, q = 0.0048; right: *r* = − 0.50, q = 0.0229), indicating reduced parahippocampal activation with increasing memory intensity. Each dot represents an individual participant; solid lines denote linear fits

After controlling for multiple comparisons using FDR correction, significant brain-behavior correlations were observed only during the low-intensity familiar odor condition. Specifically, memory intensity ratings were negatively correlated with %SC in the left parahippocampal gyrus (*r* = − 0.57, *q* = 0.0048) and the right parahippocampal gyrus (*r* = − 0.50, *q* = 0.0229) (Fig. 3). These associations indicate reduced task-evoked responses in parahippocampal regions with increasing subjective memory intensity for familiar odors presented at low intensity.

No other correlations between memory intensity, smell intensity, or Total TOM scores and neural responses survived FDR correction in this condition. Furthermore, no significant brain-behavior correlations were observed for the low-intensity unfamiliar condition or for either high-intensity familiar or high-intensity unfamiliar odor conditions following FDR correction.

## Discussion

The present study examined how odor familiarity and concentration-based perceptual intensity contribute to condition-specific spatial patterns of odor-evoked brain activation during olfactory perception. Using a 2 × 2 fMRI design with behavioral ratings, we observed that odor-evoked activation patterns varied across familiar and unfamiliar odors presented at low and high intensity levels. Importantly, behaviorally relevant brain-behavior relationships were evident specifically for familiar odors under low-intensity conditions, suggesting a context in which neural activity may be more closely coupled to subjective odor-evoked memory experience [21]. For unfamiliar odors, the higher concentration produced only a small, nonsignificant increase in perceived intensity. This may reflect variability in individual sensitivity and the participant-specific fragrance mixtures, indicating that the concentration manipulation did not reliably translate into a perceived intensity difference for unfamiliar odors in the scanned cohort.

Behaviorally, the intensity manipulation was effective for familiar odors, as high-intensity familiar odors were perceived as more intense than their low-intensity counterparts. In contrast, memory intensity ratings did not differ significantly across intensity or familiarity conditions, suggesting that subjective memory vividness may reflect relatively stable associative representations rather than immediate perceptual strength alone [5]. This dissociation between perceived odor intensity and odor-evoked memory intensity is consistent with prior work indicating that odor-evoked memories can remain vivid even when perceptual cues are weak, particularly for familiar stimuli [14].

At the neural level, all odor conditions elicited activation across primary olfactory, limbic, and associative regions, including the piriform cortex, amygdala, parahippocampal gyrus, orbitofrontal cortex, anterior cingulate cortex, and insula (Supplementary File 1). These findings are consistent with established models of olfactory processing that emphasize integration between sensory, affective, and memory-related systems [17, 27]. However, the condition-wise one-sample activation maps should be interpreted descriptively, as they indicate task-related activation within each condition but do not directly test differences between conditions.

Direct between-condition contrasts provided more specific evidence, with suprathreshold differences observed only for selected comparisons. Low-intensity unfamiliar odors showed greater activation than low-intensity familiar odors in frontal and parietal regions, consistent with greater evaluative or attentional processing when an odor cannot be readily matched to established odor representations [9, 12, 32, 36]. In addition, low-intensity unfamiliar odors showed greater activation than high-intensity unfamiliar odors in precuneus and supramarginal/angular regions, suggesting that weak and unfamiliar odor input may require greater attentional or associative evaluation. Together, these findings indicate that differences in odor-evoked activation were condition-specific rather than broadly present across all familiarity and intensity contrasts [31]. Neural differences were most evident when unfamiliar odors were presented at low intensity, supporting the interpretation that weak unfamiliar odor input may require additional evaluative processing because the stimulus cannot be readily linked to prior odor experience. In contrast, stronger odor intensity or greater familiarity may provide sufficient sensory or mnemonic information, thereby reducing the need for additional fronto-parietal recruitment.

Crucially, significant brain-behavior correlations survived FDR correction only in the low-intensity familiar condition. In this condition, memory intensity ratings were negatively correlated with percent signal change in the left and right parahippocampal gyrus, whereas no significant associations were observed with perceived odor intensity ratings or TOM scores. The selectivity of this effect suggests that familiar odors presented at low intensity may place greater reliance on memory-based representations [6], allowing individual differences in subjective memory experience to relate to medial temporal lobe activity [23].

This negative association does not indicate that stronger memory experience was accompanied by greater parahippocampal activation. Rather, stronger subjective memory intensity was associated with reduced task-evoked parahippocampal response. One possible explanation is that familiar odors capable of evoking stronger memories may be processed through more efficient access to established odor-memory representations, requiring less additional parahippocampal recruitment during passive perception. Conversely, lower memory intensity for familiar low-intensity odors may require greater contextual or associative search, leading to relatively stronger parahippocampal engagement. This interpretation is consistent with prior work showing that odor familiarity, identifiability, and recognition memory are closely related [33]. It is also broadly consistent with evidence that olfactory cues can engage medial temporal and memory-related systems, although the present findings should be interpreted cautiously because the current task involved passive odor perception and subjective memory-intensity ratings rather than an explicit recognition-memory or match/non-match task [40, 46]. Similarly, although olfactory-hippocampal connectivity has been reported in resting-state recordings, such findings should be considered supportive background rather than direct evidence for the present task-evoked parahippocampal effect [48].

The absence of significant brain-behavior correlations in primary olfactory cortex is also informative. Primary olfactory regions, including the piriform cortex, are central to odor sensory encoding and odor identity representation, whereas the present behavioral effect was specific to subjective memory intensity. Therefore, the null findings in primary olfactory cortex may suggest that individual differences in odor-evoked memory experience were not primarily driven by early sensory odor responses, but instead depended more strongly on higher-order medial temporal and associative regions involved in contextual and mnemonic aspects of odor perception. This interpretation is consistent with human olfactory neuroimaging models distinguishing early olfactory sensory regions from higher-order limbic and memory-related regions [47], and with recent single-neuron evidence showing regionally distinct odor coding in the human brain, including amygdala responses related to odor valence/preference and hippocampal activity more closely associated with odor identification [21].

Notably, comparable brain-behavior relationships were absent for unfamiliar odors and for high-intensity conditions. For unfamiliar odors, the lack of established associative representations may limit engagement of memory networks [1, 3, 17], even when perceptual intensity varies [8, 25]. For high-intensity odors, stronger sensory-driven activation may dominate neural responses, potentially masking subtler individual differences related to memory experience. Together, these findings suggest that odor familiarity and concentration-based intensity contribute to odor-evoked brain activation in a condition-dependent manner. Familiar low-intensity odors may represent a perceptual condition in which weak sensory evidence is supported by prior odor-memory representations, allowing subjective memory experience to relate more clearly to medial temporal responses.

Several limitations should be considered when interpreting these findings. First, the sample consisted exclusively of young women, which may limit generalizability given known sex differences in olfactory perception and memory [24]. Second, Other perceptual dimensions, including pleasantness, valence, trigeminal qualities, and attentional capture, may also influence odor-evoked activation and should be measured or modeled in future work. Third, the odorants were complex perfume-based fragrance mixtures and familiar and unfamiliar odors were selected individually based on subjective familiarity ratings. This approach increased ecological validity and ensured participant-specific familiarity categories, but it also limits interpretation of the findings as odor identity was not identical across familiarity conditions.

Fourth, respiration was not recorded, and odor delivery was not respiration-triggered, potentially introducing variability in odor sampling. The absence of respiratory recordings also prevented inclusion of physiological regressors to account for breathing-related BOLD fluctuations [2]. Future studies should incorporate respiratory monitoring and physiological noise correction.

Finally, although ROI-based analyses enhanced sensitivity to theoretically motivated effects, future studies could employ multivariate or connectivity-based approaches to better capture distributed network dynamics underlying familiarity- and intensity-related odor processing. Longitudinal designs may also help clarify how unfamiliar odors become familiar over time and how this transition alters brain-behavior coupling.

## Conclusion

This study demonstrates that behaviorally meaningful neural correlates of odor-evoked memory were most evident when familiar odors were presented under low-intensity conditions. Specifically, stronger subjective memory intensity was associated with reduced task-evoked activation in the bilateral parahippocampal gyrus, indicating a condition-specific relationship between odor-evoked memory experience and medial temporal responses rather than a general increase in neural activation. These findings emphasize the importance of considering both prior odor experience and concentration-based perceptual intensity when examining olfactory brain responses. More broadly, the results suggest that medial temporal memory systems may contribute to subtle, experience-dependent aspects of odor perception, particularly when familiar odors are perceived under weak sensory input conditions.

## Supplementary Information

Below is the link to the electronic supplementary material.

Supplementary Material 1

## Data Availability

Data generated and analyzed are not publicly available due to subject’s confidentiality. It will be available from the corresponding author on request.
