# Supplementary material for: Familiarity and concentration- based intensity shape spatial patterns of odor-evoked brain activation
Source: Behav Brain Funct. 2026 Nov 5;22:55. doi: 10.1186/s12993-026-00375-5 (PMC13749890; doi:10.1186/s12993-026-00375-5)
Supplement: Supplementary file 1 [file 12993_2026_375_MOESM1_ESM.docx]

**Whole-brain neural responses to familiar and unfamiliar odors at low and high salience**

**Low-Salience Familiar Odors**

| **Cluster Index** | **Voxels** | **Z-MAX** | **X (mm)** | **Y (mm)** | **Z (mm)** | **Brain Region (Hemisphere)** |
| --- | --- | --- | --- | --- | --- | --- |
| 49 | 140 | 4.62 | −18 | −4 | −14 | Left Amygdala / Piriform Cortex |
| 48 | 130 | 4.51 | 24 | 32 | 14 | Right Inferior Frontal Gyrus (BA45/47) |
| 47 | 120 | 3.80 | 40 | −82 | −18 | Right Occipital Fusiform Gyrus / Cerebellum (Crus I) |
| 46 | 97 | 4.00 | 46 | 42 | 10 | Right Middle Frontal Gyrus (DLPFC) |
| 45 | 86 | 3.63 | −2 | −86 | −24 | Midline Cerebellar Vermis / Occipital Lobe |
| 44 | 74 | 4.08 | 18 | −4 | −16 | Right Amygdala / Parahippocampal Gyrus |
| 43 | 59 | 3.65 | −22 | 34 | 14 | Left Orbitofrontal Cortex (BA11) |
| 42 | 51 | 4.01 | 64 | −26 | −10 | Right Superior Temporal Gyrus |
| 41 | 50 | 3.66 | −40 | −72 | −28 | Left Cerebellum (Crus II) |
| 40 | 48 | 3.61 | 46 | 28 | 32 | Right Dorsolateral Prefrontal Cortex (BA9/46) |
| 39 | 35 | 3.44 | −4 | −34 | 30 | Midline Cingulate Gyrus / Precuneus |
| 38 | 31 | 3.91 | −26 | −26 | 28 | Left Insular Cortex |
| 37 | 28 | 3.71 | −12 | −52 | 28 | Left Precuneus / Posterior Cingulate |
| 36 | 26 | 3.53 | 68 | −42 | −2 | Right Middle Temporal Gyrus |
| 35 | 23 | 3.45 | 2 | −52 | −8 | Midline Lingual Gyrus / Cerebellar Culmen |
| 34 | 21 | 3.97 | 20 | −22 | −12 | Right Parahippocampal Gyrus / Amygdala |

**Low-Salience Unfamiliar Odors**

| **Cluster Index** | **Voxels** | **Z-MAX** | **X (mm)** | **Y (mm)** | **Z (mm)** | **Brain Region (Hemisphere)** |
| --- | --- | --- | --- | --- | --- | --- |
| 61 | 921 | 4.85 | −32 | 56 | 16 | Left Middle Frontal Gyrus (DLPFC) |
| 60 | 712 | 4.88 | 0 | 20 | 46 | Medial Superior Frontal Gyrus / Pre-SMA |
| 59 | 557 | 4.54 | −48 | 18 | 36 | Left Inferior Frontal Gyrus (BA9/44) |
| 58 | 538 | 4.75 | 32 | 60 | 12 | Right Superior Frontal Gyrus |
| 57 | 368 | 4.68 | 40 | −62 | −22 | Right Fusiform Gyrus / Cerebellum (Crus I) |
| 56 | 354 | 4.38 | −4 | −70 | 44 | Midline Precuneus / Superior Parietal Lobule |
| 55 | 330 | 4.01 | −24 | −80 | −16 | Left Occipital Fusiform Gyrus / Cerebellum |
| 54 | 269 | 4.39 | 44 | 18 | −8 | Right Insula / Orbitofrontal Cortex |
| 53 | 238 | 4.33 | 8 | −80 | −30 | Midline Cerebellum (Crus II) |
| 52 | 179 | 4.41 | −48 | 20 | −8 | Left Insular Cortex / Frontal Operculum |
| 51 | 144 | 3.96 | 36 | −58 | 50 | Right Superior Parietal Lobule |
| 50 | 137 | 3.72 | −38 | −48 | 48 | Left Inferior Parietal Lobule |
| 49 | 114 | 4.09 | −4 | −24 | 32 | Midline Cingulate Gyrus |
| 48 | 87 | 3.85 | 52 | 24 | 38 | Right Middle Frontal Gyrus |
| 47 | 66 | 4.53 | −36 | −48 | −52 | Left Cerebellum (Lobule VI) |
| 46 | 46 | 3.56 | 10 | −86 | −8 | Right Lingual Gyrus / Occipital Lobe |
| 45 | 45 | 3.82 | −8 | 0 | 12 | Left Caudate / Ventral Striatum |
| 44 | 33 | 3.48 | −12 | −84 | −28 | Left Cerebellum (Crus II) |
| 43 | 31 | 3.57 | 38 | 36 | 20 | Right Inferior Frontal Gyrus (BA45) |
| 42 | 28 | 3.68 | −6 | −96 | −4 | Left Occipital Cortex (Lingual Gyrus) |
| 41 | 27 | 3.69 | −2 | −36 | 24 | Midline Posterior Cingulate Cortex |
| 40 | 25 | 3.40 | −44 | 36 | 22 | Left Middle Frontal Gyrus |
| 39 | 20 | 3.78 | −28 | 30 | 8 | Left Inferior Frontal Gyrus (BA44) |

**High-Salience Familiar Odors**

| **Cluster Index** | **Voxels** | **Z-MAX** | **X (mm)** | **Y (mm)** | **Z (mm)** | **Brain Region (Hemisphere)** |
| --- | --- | --- | --- | --- | --- | --- |
| 24 | 148 | 4.14 | −28 | 36 | −10 | Left Inferior Frontal Gyrus (BA44/45) |
| 23 | 106 | 4.48 | 10 | −78 | −30 | Right Cerebellum (Crus II) |
| 22 | 68 | 3.92 | 44 | −68 | −38 | Right Cerebellum (Lobule VI) |
| 21 | 55 | 3.99 | −26 | −70 | −40 | Left Cerebellum (Lobule VI) |
| 20 | 42 | 4.02 | 4 | 0 | 12 | Midline Caudate / Anterior Cingulate Cortex |
| 19 | 38 | 3.82 | −10 | −80 | −34 | Left Cerebellum (Crus II) |
| 18 | 34 | 3.94 | 32 | 34 | −10 | Right Inferior Frontal Gyrus (BA47) |
| 17 | 24 | 4.05 | −8 | −80 | −22 | Left Cerebellum (Lobule VII) |

**High-Salience Unfamiliar Odors**

| **Cluster Index** | **Voxels** | **Z-MAX** | **X (mm)** | **Y (mm)** | **Z (mm)** | **Brain Region (Hemisphere)** |
| --- | --- | --- | --- | --- | --- | --- |
| 67 | 372 | 4.28 | −28 | −70 | −38 | Left Cerebellum (Lobule VI / Crus I) |
| 66 | 282 | 4.95 | −6 | 20 | 48 | Midline Supplementary Motor Area |
| 65 | 188 | 4.70 | 20 | −14 | 30 | Right Insula / Frontal Operculum |
| 64 | 181 | 4.07 | 34 | −64 | −38 | Right Cerebellum (Lobule VI) |
| 63 | 150 | 4.19 | 40 | 26 | 2 | Right Inferior Frontal Gyrus (BA45) |
| 62 | 146 | 3.86 | −46 | 20 | −6 | Left Insular Cortex |
| 61 | 137 | 4.17 | −46 | 30 | 26 | Left Middle Frontal Gyrus |
| 60 | 135 | 4.14 | 30 | −46 | 22 | Right Inferior Parietal Lobule |
| 59 | 125 | 3.96 | 18 | 20 | 20 | Right Caudate / Prefrontal Region |
| 58 | 119 | 4.24 | 2 | 0 | 10 | Midline Anterior Cingulate Cortex |
| 57 | 85 | 4.24 | −20 | 28 | 12 | Left Superior Frontal Gyrus |
| 56 | 46 | 3.41 | 48 | 16 | 50 | Right Middle Frontal Gyrus (DLPFC) |
| 55 | 39 | 3.41 | 44 | 34 | 38 | Right Superior Frontal Gyrus |
| 54 | 33 | 3.79 | −44 | −56 | 54 | Left Superior Parietal Lobule |
| 53 | 31 | 3.71 | 42 | −74 | −30 | Right Cerebellum (Crus II) |
| 52 | 25 | 3.46 | 56 | 18 | 4 | Right Precentral Gyrus |
| 51 | 24 | 3.41 | −46 | −42 | 42 | Left Inferior Parietal Lobule |
| 50 | 23 | 3.69 | 8 | −82 | −32 | Midline Lingual Gyrus / Occipital Cortex |
| 49 | 21 | 3.60 | 24 | −28 | 30 | Right Postcentral Gyrus |
